# Abuses of the Eye

**Published:** 1872-07

**Authors:** 


The Bistoury.

ELMIRA, N. Y., JULY, 1872.

The BISTOURY is published Quarterly, upon the 1st of April, July, October and January, at Fifty Cents a year, in advance.

### ABUSES OF THE EYE.

Vision becomes impaired through a series of causes imperceptable to a careless or thoughtless observer. Imperfect vision is more common than is generally supposed. During an experience of twelve years in the examination of the eye, we have learned that persons in the possession of normal vision are comparatively few; and this indeed is not to be wondered at, when we are brought to examine the multiplicity of causes which conspire to annoy and fatigue the eye. When we consider the immense and varied amount of labor, and the abuses that it is subjected to, the wonder is, not that there are so many defective, diseased, and blind eyes, but that there is not a multitude more of them.

People are suffering from impaired vision, or diseased eyes, every blessed day, without ever once thinking of the cause, or seemingly careing about knowing. The idea never enters their stupid heads that they are doing ought to bring such infirmities upon themselves, and they survey the ophthalmic surgeon with distrust if not contempt for pointing out the possible causes for difficulty of vision, and attributing it to their bad behavior.

We will give a few of the most frequent causes for impaired vision, which the ophthalmic surgeon is called upon daily to practice for, and which not unfrequently lead to total and often incurable blindness. They are practices that are thoughtlessly persisted in, by adults, even when warned to desist by pain and weeping of the over-taxed organs; while children practice them from lack of education or instruction from parents or medical advisers.

How often we find persons who are convalesing from a severe illness, boulstered up in their beds trying to relieve the tedium of passing time by reading the morning paper or the latest novel; yes, and reading more constantly even than when in robust health. It would seem that no one of ordinary sense should be told that such a practice is very wrong, that it inflicts a serious injury to the nervous power of the eye, and that partial blindness often ensues, from conjestion of the retina, or nerve of the eye;—yet, are we called upon, almost daily, to teach this lesson.

A young lady—or old man, for that matter—is engaged in reading a very entertaining book; twilight steals on, almost imperceptibly, yet the reading is continued in, the eye strained for page after page, until it becomes impossible to distinguish the letters longer, when the book is reluctantly thrown down, and the eyes pressed between finger and thumb to relieve them of a painful and tired feeling. Surely it is not necessary to inform that young lady or old gentleman that they are abusing their eyes, and that such habits will surely lead to serious disturbance of the functions of the eye.

Travelers in railroad cars, supply themselves with magazines, by the reading of which they try to relieve the ennui of the journey.—The constant oscillation of the car varies the distance between the book and eye, causing a constant effort at accommodation of the organ to the varying distance. From this reason, the person is unable to read a great while at a time without experiencing great weariness of the eye, heaviness of the eye-lids, and a disposition to sleep. Should the reading be persisted in, paralysis of accommodation ensues, causing blurred and indistinct vision, from which the eye often never recovers.

The persistent use of the eye by artificial light gives rise to a variety of difficulties of vision.—Watchmakers, seamstresses, tailors, engravers, students, and literary men, who are in the habit of using their eyes by dim or flickering lights, or yet by brilliant gas light, soon suffer from impaired vision. Constantly changing lights are also wearisome to the eye, as when reading under a tree, where from the motion of the leaves, lights and shadows constantly chase each other over the page of the book, wearying the *iris* and *retina* by not affording them sufficient time in which to adapt themselves to these sudden changes. In all cases, for the preservation of sight, the source of light should come from over the shoulder, should be steady, and sufficiently bright to render the object observed perfectly distinct. Never allow the light to come between the eye and book, or from in front of you. Always sit with your back to the window or gasjet when reading or sewing.

The great majority of every community, when they find their eyes failing them, from whatever cause, generally resort to the spectacle shop or jeweller to seek aid in glasses. Jewellers and spectacle venders as a rule, are not, nor are they expected to be, scientific opticians, nor yet acquainted with the manifold defects of vision.—Therefore, when the patient applies for glasses, he is handed a tray of spectacles, from which he is allowed to select such as magnify the object sufficiently to be seen, or such as may suit the notions of the customer. Such persons, being ignorant of what is required, or what the *cause* of the defect in vision may be, *adapt their eyes to the spectacles*, instead of doing precisely the reverse.

As a rule, no person should ever wear glasses without first consulting a competent oculist, who should test the eyes, see what is required in the shape of a lens, and then recommend the person to some one skilled in mounting and adjusting such lenses. In testing the eyes for lenses, it is often found that each eye requires an entirely different lens in order to afford the patient useful vision. Often lenses have to be ground of a peculiar form to accommodate an imperfect eye. In such cases, the patient may try on all the spectacles in his jeweler's establishment, without finding a pair that benefits his vision in the least.

Again, the eye is often abused by an exactly contrary course; many persons supposing, or pretending to, that it is proper to go without glasses as long as possible, straining their eyes in the vain effort to see objects at a distance for which they have lost their accommodation.—Whenever a person cannot see to read at the usual distance without the eye becoming fatigued or painful, while the letters become indistinct and blurred, he should at once procure the proper spectacles suited to the condition of his eyes, by which course he will preserve his vision to a much longer period.

These are a few of the most prominent abuses to which our eyes are subjected. We hope by calling the attention of our readers to them, that they may be led to avoid them, and so preserve their eyes, with perfect vision, to a good old age.

